# Pediatric obstructive sleep apnea-related risk factors: A cross-sectional study

**DOI:** 10.2340/aos.v83.41385

**Published:** 2024-09-09

**Authors:** Júlia Machado Saporiti, Deborah Castagno, Clarissa Delpizzo Castagno, Maria Perpétua Mota Freitas, Marília Leão Goettems, Noéli Boscato

**Affiliations:** a School of Dentistry, Federal University of Pelotas, Pelotas, Brazil; b Clinical Practice at Pelotas Sleep Institute, School of Dentistry, Federal University of Pelotas, Pelotas, Brazil; c Clinical Practice at Pelotas Sleep Institute, Otorhinolaryngology and Member of the Brazilian Sleep Association, Pelotas, Brazil; d School of Dentistry, Graduate Program in Dentistry, Lutheran University of Brazil, Canoas, Brazil; e School of Dentistry, Graduate Program in Dentistry, Federal University of Pelotas, Pelotas, Brazil

**Keywords:** Respiration disorders, sleep apnea syndromes, pediatric, sleep monitoring, obesity

## Abstract

**Objectives:**

This study investigated obstructive sleep apnea (OSA)-related risk factors in children and adolescents.

**Materials and methods:**

Records of 187 subjects from a private medical clinic were reviewed. Overnight polysomnography recordings and self/parent reports were gathered. Descriptive analysis of sociodemographic, anthropometric, sleep quality and sleep architecture variables and OSA diagnosis were performed. Associations between independent variables and OSA diagnosis were assessed through multivariable logistic regression with robust variance, with a significance level of 5%.

**Results:**

132 participants were diagnosed with OSA, and 55 were classified as “no OSA” (29.41%). Those overweight or obese were 4.97 times more likely to have OSA than those with normal weight (*P =* 0.005). Those who reported loud snoring were 2.78 times more likely to have OSA than those who reported mild or moderate snoring intensity. A one-unit increase in arousal index leads to 1.39 increase in the odds ratio (OR) of individuals diagnosed with OSA (*P* < 0.001), and each one-unit increase in sleep efficiency leads to 1.09 higher odds of not having OSA (*P* = 0.002).

**Conclusions:**

Significantly increased OSA-related risk factors among overweight/obese children and adolescents and among those who had a parental/self-report of loud snoring were found.

## Introduction

Pediatric Obstructive Sleep Apnea (OSA) is a respiratory disorder characterized by obstructive upper airway events that disrupt normal ventilation during sleep, resulting in sleep fragmentation and non-restorative sleep [1]. It is a common and potentially serious condition among Sleep-Related Breathing Disorders (SRBD), leading to cardiovascular, cognitive, and behavioral complications, increasing children’s morbidity, and impairing quality of life [1]. OSA prevalence among children and adolescents ranges from 0.1 to 13% [2–5]. Nevertheless, studies have reported a greater prevalence, from 20 to 60.7%, in overweight or obese children and adolescents [6, 7], while contradictory findings have been reported regarding OSA prevalence among genders [2–6, 8].

OSA diagnosis’s gold standard is through nocturnal laboratory-based polysomnography (PSG) with objective measurements, specific cut-offs, and severity assessment [1, 9]. According to the pediatric diagnostic criteria stated by American Academy of Sleep Medicine (AASM) to confirm the OSA diagnosis in individuals younger than 18 years, the presence of one or more OSA-related signs and/or symptoms must be combined with at least one obstructive/mixed apnea or hypopnea episode per hour of sleep demonstrated by PSG [10]. However, most studies have reported OSA diagnosis from questionnaires rather than overnight PSG data, although interviews do not present enough accuracy to establish OSA diagnosis. Failure to recognize OSA-related signs and symptoms precludes early treatment [11]. Therefore, this cross-sectional study investigated OSA-related risk factors in children and adolescents who had undergone nocturnal laboratory-based PSG. The hypothesis tested was that there will be an association between OSA and sex, overweight/obesity, poor sleep quality, reduced sleep efficiency, and greater arousal index.

## Materials and methods

### Study design, setting, and participants

This cross-sectional clinical study was approved by the institutional Human Research Ethics Committee (protocol: 84221418.4.0000.5318), reported according to the Strengthening the Reporting of Observational Studies in Epidemiology (STROBE) [12]. This study included participants who were available to participate, diagnosed with OSA by nocturnal PSG and aged 1–18 years old (children, aged 1–11 years, and adolescents, aged 12–19 years). Written consent was obtained from parents and/or legal guardians and verbal assent from participants aged more than 12 years. Data from questionnaires and overnight PSG data obtained at a private medical outpatients’ sleep clinic from January 2012 to December 2017 were assessed and reviewed. We assigned a code to each participant to guarantee the anonymity and confidentiality of the data. Individuals with a history of neuromuscular disease (e.g., conditions such as cerebral palsy or muscular dystrophy); craniofacial syndrome (e.g., Pierre Robin sequence, craniosynostosis, brachycephaly); genetic syndromes (e.g., Down, Prader-Willi); or other comorbidities such as allergic rhinitis, adenotonsillar hypertrophy, obesity, endocrine disorders (e.g., hypothyroidism or acromegaly), gastroesophageal reflux disease and participants for whom we did not obtain signed informed consent were excluded.

### Data collected

PSG recordings in combination with data from a self-administered or parental-reported questionnaire were collected. The questionnaires were answered immediately before the PSG exam by the children’s parents (when the child was less than 12 years old) or by the adolescents, assisted by their parents.

### Sociodemographic and anthropometric data

Sociodemographic data [including gender (male or female); age group (children or adolescents); parental educational level (<8 years or ≥8 years), family structure (nuclear or non-nuclear)], anthropometric data [height and weight] and lifetime history were gathered. The height and weight data were either self-reported or reported by parents based on physical examination with documentation of anthropometric measurements weight (Kg), height (m), BMI (Kg/m^2^) collected in previous medical appointments. The BMI percentile (BMI%) for each participant aged 2 and older was calculated based on growth charts, and the patients’ weight group was defined as “Normal weight” when BMI% was between 5^th^ percentile to less than the 85^th^ percentile; “Overweight” when BMI% was at or above the 85^th^ percentile and below the 95^th^ percentile; and “Obese” when BMI% was above the 95^th^ percentile. While for participants under 2 years old, obesity was calculated using weight-for-length and weight-for-age. Weight-for-length compares a child’s weight to their length, while weight-for-age compares it to the average weight of children of the same age and sex. These measures are plotted on growth charts specific to the child’s age and sex. If the weight exceeds the 95^th^ percentile, it may indicate obesity, prompting recommendations for dietary changes, increased physical activity, and regular growth monitoring [13].

### Sleep quality via CSHQ

Sleep quality was evaluated with a questionnaire based on the Children’s Sleep Habits Questionnaire (CSHQ) [14], applied using closed questions as follows: Sleep behavior: snoring during the night, gasping or difficulty breathing during the night, snoring intensity, waking up more than twice a night, sleep enuresis; Morning wake-up: sleepiness, mouth breathing during the day, learning problems, and relationship difficulty. The questions were dichotomous (no or yes), except for snoring intensity, which was evaluated in categories (mild, moderate, or loud).

### OSA diagnosis via PSG

According to the AASM International Classification of Sleep Disorders (ICSD-3) guidelines [10], the OSA diagnosis, sleep stages, and related respiratory events were defined. Children and adolescents slept for one night in nocturnal laboratory-based polysomnography (PSG). PSG recordings were performed with Alice 5 PSG system (Philips Respironics, USA), including electroencephalography (EEG) at F3-M2, F4-M1, C3M2, C4-M1, O1-M2, and O2-M1; electrooculography (right and left eyes); 3 derivations of electrocardiography (ECG); electromyography (EMG) of the chin and masseter muscles for repetitive masticatory muscle activity (RMMA) scoring, and of the anterior tibialis (bilateral) for scoring limb movements. Audio/video recordings were made and used to interpret OSA findings. Sleep breathing assessment included oronasal airflow monitoring using a thermistor channel, plethysmography to track thoracic and abdominal movements, body position sensors to record body posture, oxygen saturation, and heart rate monitoring via pulse oximetry, snoring and airflow measurement through pressure transducer channels in nasal prongs, and continuous transcutaneous carbon dioxide (CO^2^) monitoring. The relative duration of each sleep stage was expressed as a percentage (%) of the total sleep time (TST). We defined the apnea index (AI) and the apnea-hypopnea index (AHI) as the number of apneas and the number of apneas plus hypopneas per hour of sleep, respectively. Apneas was defined as a complete airflow cessation for at least two breath cycles, and hypopneas was a reduction in airflow by at least 30% for at least two breath cycles, with accompanying oxygen desaturation of 3% or more or arousal. Additionally, we calculated the obstructive AHI, and the respiratory disturbance index (RDI), which includes the number of apneas, hypopneas, and respiratory effort-related arousals (RERAs) per hour of sleep. Arousals from sleep are scored based on specific criteria, including duration and frequency following [10, 15]. Sleep stages are scored according to standard criteria including wakefulness, non-rapid eye movement (NREM) sleep stages N1, N2, N3, and rapid eye movement (REM) sleep. PSG recordings were scored by a sleep technician and confirmed by an otorhinolaryngologist (C.D.C). According to ICSD-3 guidelines [10] the criteria A and B must be met for OSA diagnosis: (A) Participants must self-report a positive answer for at least one of the following signs and symptoms: snoring during the night; labored, paradoxical, gasping, or difficulty breathing during the night; sleepiness; learning problems; hyperactivity, behavioral problems in combination with (B) the PSG exam demonstrating one or more obstructive apneas, mixed apneas, or hypopneas per hour of sleep [10]. According to AASM, including the Manual for the Scoring of Sleep and Associated Events [10], pediatric criteria for OSA apply to patients younger than 18 years, However, for PSG scoring, adult diagnostic criteria may be used for patients aged 13 to 18 years. In our study, children with an AHI ≥ 1 were diagnosed with OSA [10], and its severity was classified as mild (AHI ≥ 1 and <5 events/h), moderate (AHI ≥ 5 and <10 events/h), and severe OSA (AHI ≥ 10 events/h). The OSA diagnosis in adolescents was aligned with the AASM criteria for adults as follows: mild (AHI ≥ 5 and < 15 events/h), moderate (AHI ≥ 15 and < 30 events/h), and severe OSA (AHI ≥ 30 events/h) [10]. However, although the same criteria may be used for adolescents and adults, physicians should evaluate each case individually, taking into account age-specific and developmental factors [16].

### SB detection via PSG

The “definite sleep bruxism (SB)” was defined based on standard nocturnal laboratory-based PSG proposed by Lobbezoo and collaborators [17]. Participants were diagnosed with SB (yes or no) if the electromyography recordings of the RMMA index were greater than two episodes per hour of sleep [17].

### Sleep architecture

The sleep architecture parameters were scored and reported in minutes. The sleep onset latency is the period between the beginning of the PSG recording and the first episode scored as sleep, the sleep onset [18]. The rapid-eye-movement (REM) sleep latency is the time from the beginning of the PSG recording and the first epoch of REM sleep [16]. Wake after sleep onset (WASO) includes periods of wakefulness after sleep onset [15]. TST is the total time asleep scored during recording [18]. Stages N1, N2, and N3 represent the time spent in each non-rapid eye movement sleep (NREM), and Stage R represents the REM sleep [15]. Sleep efficiency was defined as the percentage of the total recording time (TRT) spent asleep. A sleep efficiency ≥ 85% was considered an adequate indicator of sleep quality [19]. The arousal index is defined as the number of sleep arousals per hour of the TST [20].

### Sample size

Sample size calculation was performed considering an alpha error of 5% and a study power of 80% [2]. The minimum sample size to satisfy the requirements was estimated at 71 individuals. To account for potential losses during the experiment, 277 PSG records and questionnaires were evaluated, and finally, 187 met the inclusion criteria.

### Statistical methods

The statistical software program Stata 14.0 (Stata Corp, College Station, USA) was used for all analyses. Categorical variables were reported as frequencies and percentages, and continuous data as means and standard deviations (SD).

Analyzes were performed considering OSA diagnosis (dichotomized: no OSA or OSA) as the primary outcome. Multivariable logistic regression with robust variance was used to assess the associations between the independent variables (sociodemographic, anthropometric, sleep quality, sleep architecture, and SB) and OSA diagnosis, estimating odds ratios (OR) and 95% confidence intervals (CI). All variables with a *P-*value <0.20 in the unadjusted analysis were considered potential confounders and included in the multivariable analysis. The significance level for all analyses was set at 5%.

## Results

Table 1 shows the descriptive analysis of OSA diagnosis according to sociodemographic and anthropometric data. 187 children and adolescents were included in this study. Ninety-five were males (50.80%), children’s ages ranged from 1 to 11 years (6.68 ± 2.51), and adolescents’ ages from 12 to 18 years (14.22 ± 1.62), with a mean age of 8.86 ± 4.12 years. Eighty-five participants (54.14%) were overweight or obese, with similar distribution between children (53.33%) and adolescents (55.77%). One hundred thirty-two participants were diagnosed with OSA (70.59%); among these, 92 (69.70%) were children and 40 (30.30%) adolescents. The mean age was 8.95 ± 4.29, and the majority were males (54.55%).

**Table 1:** Descriptive analysis of OSA diagnosis and sociodemographic and anthropometric data in children and adolescents (*n* = 187).

Sociodemographic	No OSA (*n* = 55)	OSA (*n* = 132)	Total (*n* = 187)
**Gender, *n* (%)**			
Female	32 (58.18)	60 (45.45)	92 (49.20)
Male	23 (41.82)	72 (54.55)	95 (50.80)
**Age group, *n* (%)**			
Children (1–11 years)	41 (74.55)	92 (69.70)	133 (71.12)
Adolescents (12–19 years)	14 (25.45)	40 (30.30)	54 (28.88)
**Parental educational level, *n* (%)**			
<8 years	6 (12.24)	9 (7.56)	15 (8.93)
≥8 years	43 (87.76)	110 (92.44)	153 (91.07)
**Family structure, *n* (%)**			
Nuclear	38 (74.51)	84 (67.20)	122 (69.32)
Non-nuclear	13 (25.49)	41 (32.80)	54 (30.68)
**ANTHROPOMETRIC**			
**Weight group, *n* (%)**			
Normal	26 (61.90)	46 (40.00)	72 (45.86)
Overweight/Obese	16 (38.10)	69 (60.00)	85 (54.14)

OSA: Obstructive Sleep Apnea.

Values are expressed as frequencies and percentages.

Values different from 187 (total of participants) and 132 (OSA) are due to missing responses.

Table 2 shows the descriptive analysis of OSA diagnosis according to sleep quality variables and Table 3 according to polysomnographic parameters of sleep architecture. Overall, the severe OSA group presented higher mean values of sleep onset latency, REM sleep latency, WASO, stages N1, N2, R, and arousal index than those with no OSA. A decrease in TST, Stage N3, and sleep efficiency was observed between the individuals with no OSA and those with OSA. A higher sleep efficiency (≥ 85%) was observed in individuals with no OSA.

**Table 2:** Descriptive analysis of OSA diagnosis and sleep quality variables based on nocturnal laboratory-based polysomnography recordings in children and adolescents (*n* = 187).

	No OSA (*n* = 55)	OSA (*n* = 132)	Total (*n* = 187)
**Sleep behavior**			
**Snoring during the night**			
No	13 (23.64)	7 (5.43)	20 (10.87)
Yes	42 (76.36)	122 (94.57)	164 (89.13)
**Snoring intensity**			
Mild	19 (43.18)	34 (28.81)	53 (32.72)
Moderate	15 (34.09)	34 (28.81)	49 (30.24)
Loud	10 (22.73)	50 (42.38)	60 (37.04)
**Gasping or difficulty breathing during the night**			
No	22 (40.74)	38 (29.23)	60 (32.61)
Yes	32 (59.26)	92 (70.77)	124 (67.39)
**Waking up more than twice a night**			
No	13 (24.53)	35 (27.13)	48 (26.37)
Yes	40 (75.47)	94 (72.87)	134 (73.63)
**Sleep enuresis**			
No	42 (76.36)	101 (77.69)	143 (77.30)
Yes	13 (23.64)	29 (22.31)	42 (22.70)
**Sleep bruxism**			
No	31 (57.41)	82 (62.12)	113 (60.75)
Yes	23 (42.59)	50 (37.88)	73 (39.25)
**MORNING WAKE UP**			
**Sleepiness**			
No	22 (40.74)	41 (49.40)	82 (44.09)
Yes	32 (59.26)	42 (50.60)	104 (55.91)
**Mouth breathing during the day**			
No	12 (22.64)	21 (16.15)	33 (18.03)
Yes	41 (77.36)	109 (83.85)	150 (81.97)
**Learning problems**			
No	43 (79.63)	103 (79.78)	146 (79.78)
Yes	11 (20.37)	26 (20.16)	37 (20.22)
**Relationship difficulty**			
No	45 (83.33)	115 (89.15)	160 (87.43)
Yes	9 (16.67)	14 (10.85)	23 (12.57)

OSA: Obstructive Sleep Apnea.

Values are expressed as frequencies and percentages.

Values different from 187 (total of participants) and 132 (OSA diagnosed participants) are due to missing responses.

**Table 3:** Descriptive analysis of OSA diagnosis and sleep architecture based on nocturnal laboratory-based polysomnography in children and adolescents (*n* = 187).

Sleep architecture	No OSA (*n* = 55)	OSA (*n* = 132)
	Mean ± SD	Mean ± SD
**Sleep onset latency** (min)	36.49 ± 33.12	38.52 ± 32.34
**REM sleep latency** (min)	133.46 ± 55.62	145.18 ± 68.09
**WASO** (min)	35.34 ± 31.37	49.82 ± 47.72
**TST** (min)	394.56 ± 57.97	384.10 ± 67.51
**Stage N1** (min)	3.12 ± 1.87	4.27 ± 4.70
**Stage N2** (min)	49.52 ± 11.40	50.11 ± 9.96
**Stage N3** (min)	27.77 ± 11.15	25.96 ± 8.27
**Stage R** (min)	19.46 ± 5.83	20.47 ± 9.87
**Arousal Index**	7.51 ± 2.47	14.25 ± 14.25
**Sleep Efficiency** (%)	86.22 ± 9.10	80.50 ± 12.55

OSA: Obstructive Sleep Apnea; REM sleep latency: rapid-eye-movement sleep latency; WASO: wake after sleep onset; TST: total sleep time; Stage N1: stage 1 non-rapid eye movement sleep; Stage N2: stage 2 non-rapid eye movement sleep; Stage N3: stage 3 non-rapid eye movement sleep; Stage R: rapid-eye-movement sleep.

Values are expressed as mean and standard deviation (SD).

Table 4 presents the unadjusted and adjusted multivariable logistic regression analysis according to OSA diagnosis and sociodemographic, anthropometric, and sleep quality data. The adjusted analysis revealed that overweight/obese were 4.97 times more likely to have OSA than those in the normal weight group (OR: 4.97, 95% CI: 1.83 – 13.45; *P =* 0.002). Also, those who had a parental/self-report of loud snoring intensity were 2.78 times more likely to have OSA than those who reported mild or moderate snoring intensity (OR: 2.78, 95% CI: 1.01 – 7.67; *P* = 0.049). Among the sleep architecture variables presented in Table 5, a one-unit increase in the arousal index leads to a 1.43-fold increase in the OR of having OSA (OR: 1.43, 95% CI: 1.20 – 1.70; *P* < 0.001) and each one-unit increase in the sleep efficiency leads to 1.06 higher odds of not having OSA (OR: 0.94, 95% CI: 0.90 – 0.98; *P* = 0.003).

**Table 4:** Logistic regression analysis between OSA diagnosis and sociodemographic, anthropometric and sleep quality data based on nocturnal laboratory-based polysomnography in children and adolescents (*n* = 187).

	Unadjusted OR (95% CI)	*P*-value	Adjusted OR (95% CI)	*P*-value
**Sociodemographic**				
**Gender**				
Female	1			
Male	1.67 (0.88 – 3.16)	0.115	1.82 (0.67 – 4.97)	0.240
**Age group**				
Children	1			
Adolescent	1.27 (0.62 – 2.60)	0.507	-	-
**Parental educational level**				
<8 years	1			
≥8 years	1.71 (0.57 – 5.10)	0.339	-	-
**Family structure**				
Nuclear	1			
Non-nuclear	1.43 (0.68 – 2.97)	0.343	-	-
**Weight**				
**Weight group**				
Normal	1			
Overweight/obese	2.44 (1.18 – 5.05)	0.016	4.97 (1.83 – 13.45)	0.002*
**Sleep behavior**				
**Snoring intensity**				
Mild	1			
Moderate	1.27 (0.55 – 2.90)	0.577	1.38 (0.54 – 3.55)	0.504
Loud	2.79 (1.15 – 6.76)	0.023	2.78 (1.01 – 7.67)	0.049
**Waking up more than twice per night**				
No	1			
Yes	0.87 (0.42 – 1.83)	0.718	-	-
**Sleep enuresis**				
No	1			
Yes	0.93 (0.44 – 1.96)	0.844	-	-
**Sleep bruxism**				
No	1			
Yes	0.82 (0.43 – 1.57)	0.551	-	-
**Morning wake up**				
**Mouth breathing during the day**				
No	1			
Yes	1.52 (0.68 – 3.37)	0.304	-	-
**Relationship difficulty**				
No	1			
Yes	0.61 (0.24 – 1.51)	0.284	-	-

OSA: Obstructive Sleep Apnea.

* Statistically significant difference by adjusted logistic regression; *P* ≤ 0.05.

**Table 5:** Logistic regression analysis between OSA diagnosis and polysomnographic parameters of sleep architecture in children and adolescents (n = 187)

Sleep architecture	Unadjusted OR (95% CI)	*P*-value	Adjusted OR (95% CI)	*P*-value
**Sleep onset latency**	1.00 (0.99 – 1.01)	0.708	-	-
**REM sleep latency**	1.00 (0.99 – 1.00)	0.226	-	-
**WASO**	1.01 (1.00 – 1.02)	0.035	1.00 (0.99 – 1.01)	0.889
**TST**	1.00 (0.99 – 1.00)	0.294	-	-
**Stage N1**	1.19 (0.99 – 1.42)	0.061	1.01 (0.91 – 1.13)	0.804
**Stage N2**	1.00 (0.97 – 1.04)	0.733	-	-
**Stage N3**	0.98 (0.95 – 1.01)	0.224	-	-
**Stage R**	1.01 (0.98 – 1.05)	0.393	-	-
**Arousal Index**	1.40 (1.20 – 1.64)	<0.001	1.43 (1.20 – 1.70)	<0.001
**Sleep efficiency**	0.95 (0.92 – 0.98)	0.005	0.94 (0.90 – 0.98)	0.003

OSA: Obstructive Sleep Apnea; REM sleep latency: rapid eye-movement sleep latency; WASO: wake after sleep onset; TST: total sleep time; Stages N1, N2 and N3: stage 1, 2 and 3 non-rapid eye-movement sleep; Stage R: rapid eye-movement sleep;

*Statistically significant difference by adjusted Logistic regression; P ≤ 0.05.

## Discussion

The hypothesis of this study was partially accepted since significant associations were found between OSA diagnosis and overweight/obese children and adolescents. Reduced sleep efficiency and increased arousal index were also observed in OSA participants. The OSA prevalence was 70.59%, ranging from 69.17% in children to 74.07% in adolescents, with mild OSA diagnosis in most participants (62.88%). Similar prevalence has been observed in previous studies assessing OSA diagnosis via PSG in young children (60%) [21] and adolescents (60.5% [6] to 61%) [22]. Interestingly, epidemiological studies using questionnaires and home monitoring PSG have reported lower prevalence values, ranging from 0.1 to 13% [2–5, 23]. The use of different methodologies and AHI cut-offs could explain the wide OSA prevalence range among studies [15, 24]. In this study, children with an AHI ≥ 1 were diagnosed with OSA via PSG, and the OSA diagnosis in adolescents was aligned with the AASM criteria for adults with an AHI ≥ 5 [10]. It is important to highlight that, although the AASM criteria are the same for adolescents and adults, adolescents are in a developmental stage and may have different needs in terms of treatment and management of sleep apnea [10, 16].

Our findings showed no significant difference between genders, agreeing with previous studies [3, 5, 6]. Conversely, studies have shown a higher OSA prevalence in males [2, 8, 25]. A possible explanation is based on the more extended collapsible airway segment found in prepubertal male children [25]. Also, a protective effect of estrogen and/or progesterone against OSA in females has been suggested [26]. However, there is no consensus regarding the association between male gender and pediatric OSA yet.

Overweight/obese children and adolescents represented 54.14% of the sample and were 4.97 times more likely to have OSA than those with normal weight. Overweight/obesity were independent predictors of OSA, even after adjustment for age. Our findings are in agreement with previous studies [6, 22, 27]. A recent systematic review found a significant association between obesity and the increased AHI in children and adults, evidencing that obesity is a significant risk factor for OSA [28]. Nevertheless, it is unclear if this association is related to fat tissue deposition in the cervical area, favoring upper airway collapsibility [29], or due to higher respiratory effort performed by obese individuals [30]. Although tonsillar hypertrophy is recognized as the main childhood OSA-related risk factors [27], overweight/obesity may be a more important risk factor in the pathophysiology of OSA in adolescents [22, 24]. Further investigations are needed to clarify this association [24].

Snoring, gasping or breathing effort, frequent awakenings, and sleep enuresis have been related to OSA and poor sleep quality [1, 9]. In our study, only the snoring intensity presented a weak, although significant association after adjustment. Previous SRBD diagnosis guidelines [1, 10] report loud snoring as a recognized sign of OSA, justifying the use of questionnaires addressing snoring intensity in children and adolescents. However, no significant association between SB and OSA was found in our study. These findings differ from studies that revealed a significant association between OSA and SB among adolescents [31], and children [32, 33]. SB is likely to reestablish airway patency following the arousals caused by obstructive respiratory sleep events [34]. However, there is a lack of studies investigating the relationship between SB and OSA in children and adolescents via PSG [35].

After controlling for confounders, sleep efficiency and arousal index were significantly associated with OSA. The sleep efficiency was lower than 85% in OSA participants. Since sleep efficiency is an indicator of sleep quality [19], children and adolescents with OSA had poorer sleep quality than those with no OSA. Our results demonstrate that an increase in sleep efficiency significantly decreases the odds of having OSA. Previous studies [20, 21] have reported no significant difference in sleep efficiency between OSA and no OSA individuals. However, our findings align with an earlier study that evaluated a sample of children aged 3–5 years old and found lower sleep efficiency in those with OSA [36]. Regarding arousals, there was a greater arousal index in children and adolescents with OSA, with statistical significance, corroborating earlier studies [20, 21]. The arousals are likely to occur as a response after respiratory events, preventing sleepers from getting a night of restorative sleep, decreasing cerebral oxygenation, and leading to cognitive and physical impairments [20, 37].

Some limitations and strengths should be addressed to our study. Firstly, the sample consists of participants who had undergone PSG in a private medical outpatients’ sleep clinic, thus not representing the entire pediatric population. However, the use of data from PSG recordings is among the strengths of this study, given its accuracy in establishing OSA diagnosis [9]. Secondly, this is a cross-sectional study, and OSA-related factors might be better defined in prospective studies and longer follow-ups [8]. Nevertheless, cross-sectional epidemiological studies present adequate study design to assess the prevalence and associations, and the mandatory use of PSG for OSA diagnosis in children and adolescents precludes its use in larger samples. Some strengths may be also observed. The OSA diagnosis was based on AASM criteria. Most of the available studies used only the AHI threshold recorded in the PSG to identify OSA [6, 25, 27]. This study aimed to address OSA diagnosis based on AASM criteria, which requires both PSG typical OSA respiratory events in combination with specific signs and symptoms to meet the necessary criteria for OSA diagnosis [10]. Also, other studies used home monitoring PSG to assess OSA, which could underestimate OSA prevalence since they lack EEG to score the sleep-wake stages, and so the rate of respiratory events is calculated based on TRT rather than the actual TST [16], justifying the need for investigations using PSG.

Some questions remain unanswered regarding pediatric OSA. Further research should investigate whether gender plays a role in predicting the disease in individuals younger than 18 years, especially in children. Indeed, the relationship between OSA, bruxism, and sleep architecture should be better investigated. Overweight or obese children and adolescents experienced a higher likelihood of developing OSA than those with normal weight. Our findings also underscore the importance of parental/self-report loud snoring as an indicator of OSA-related risk factors for early management. Interestingly, those experiencing OSA had decreased sleep efficiency and increased arousal index, evidencing sleep fragmentation and non-restorative sleep. On the other hand, our data points to a potential protective factor, an increase in sleep efficiency is associated with a decreased OSA likelihood. Since at least one of the OSA-related signs and symptoms must be evaluated in combination with one or more obstructive apneas recorded by PSG, health professionals have an essential role in identifying and referring pediatric OSA for early treatment.
